# ClusterViSu, a method for clustering of protein complexes by Voronoi tessellation in super-resolution microscopy

**DOI:** 10.1038/srep24084

**Published:** 2016-04-12

**Authors:** Leonid Andronov, Igor Orlov, Yves Lutz, Jean-Luc Vonesch, Bruno P. Klaholz

**Affiliations:** 1 Centre for Integrative Biology (CBI), Department of Integrated Structural Biology, IGBMC (Institute of Genetics and of Molecular and Cellular Biology), 1 rue Laurent Fries, Illkirch, France; 2 Centre National de la Recherche Scientifique (CNRS) UMR 7104, Illkirch, France; 3 Institut National de la Santé et de la Recherche Médicale (INSERM) U964, Illkirch, France; 4 Université de Strasbourg, Strasbourg, France

## Abstract

Super-resolution microscopy (PALM, STORM etc.) provides a plethora of fluorescent signals in dense cellular environments which can be difficult to interpret. Here we describe ClusterViSu, a method for image reconstruction, visualization and quantification of labelled protein clusters, based on Voronoi tessellation of the individual fluorescence events. The general applicability of this clustering approach for the segmentation of super-resolution microscopy data, including for co-localization, is illustrated on a series of important biological objects such as chromatin complexes, RNA polymerase, nuclear pore complexes and microtubules.

Single-molecule super-resolution microscopy recently brought fluorescence imaging into unprecedented levels of details by reaching the nanometer range of localization precision^1^ and the ~30 nm range of actual resolution as estimated by Fourier ring correlation (FRC)^2^,^3^. As opposed to classical confocal microscopy, the data of stochastic super-resolution is essentially discontinuous because it comprises a set of points with molecular coordinates of the localization events, and hence it does not allow a direct segmentation. Special processing methods have been developed recently, including drift correction, visualization and estimation of co-localization^4^,^5^,^6^,^7^. However, considering the plethora of fluorescent signals in dense environments such as chromatin, the interpretation of localization data can become rather challenging with regards to image segmentation and cluster analysis. This is particularly true with respect to identifying and quantifying differently concentrated regions of labeled complexes. In some cases, super-resolution clustering on chromatin^8^, neurons, lymphocytes and cell-surface receptors^9^,^10^,^11^ has used Ripley’s analysis for global overview of cluster properties in a given region; alternatively, pair correlation analysis^12^ has been used, e.g. for studies of plasma membrane proteins^13^. For the estimation of local densities, for segmentation and cluster analysis methods such as Ripley’s L function^9^,^10^,^14^, median or Gaussian filtering of histogram images, k-means^8^ and DBSCAN^15^ clustering can provide some degree of visual clustering, but quantification is not straightforward. Recently, a Bayesian approach was developed for identification of clusters from a set of cluster proposals from Ripley’s analysis^16^. Here we describe a method that we call ClusterViSu, which is based on Voronoi diagrams and tessellation of the individual fluorescence events for visual and quantitative clustering analysis of super-resolution microscopy data. When this manuscript was under review a similar method appeared using the same concept of segmentation based on Voronoi diagrams, called SR-Tesseler^17^. While ClusterViSu comprises additional features both studies complement each other; a more detailed comparison of the two methods including similarities and differences is made towards the end of this manuscript.

A Voronoi diagram, also known as Dirichlet decomposition, is a tessellation where a tile corresponding to a given data point is a locus of all points of space closest to this data point^18^. Applications of Voronoi tessellations are found in various fields from mathematics to natural sciences^19^, e.g. for cluster detection in atom probe microscopy^20^. In the context of super-resolution microscopy as introduced here the Voronoi sites would correspond to the experimentally determined molecular coordinates of individual fluorophores. A Voronoi cell represents an area of influence of the data point it contains, and thus the local density in the proximity of a given point can be determined as the inverse of the cell area. This provides a direct precise measurement of the local density, unlike Ripley’s analysis where the result depends on the chosen search radius. The values of local densities, interpolated to a regular grid, create a density map, which can be used for a direct image reconstruction and visualization of super-resolution data in the same manner as do standard histogram^21^ or Gaussian^22^ representation modes (Figs 1 and 2). The graphical properties of Voronoi diagrams and their mathematical propensity for potential quantification calculations prompted us to develop a method for clustering analysis based on Voronoi tessellations.

To validate the usage of the Voronoi diagram concept for super-resolution imaging, we first performed comparisons of image reconstructions using histogram^21^ or Gaussian^22^, and the Voronoi representation mode introduced here. Voronoi-based visualization both reduces visible noise and emphasizes features (Fig. 1A–D), and preserves the resolution upon image rendering equally well or better as compared to histogram and Gaussian mode image representations (Fig. 1F). The reason for this is that low-density and randomly distributed signals of the noise generate huge Voronoi cells, and the corresponding density values are much lower than those of highly dense regions. On the other hand, localizations at borders of dense areas have Voronoi cells significantly bigger than those of inner localization. This effect leads to muting of border localizations compared to internal ones, which is seen as effective increase of sharpness and contrast of the reconstructed image. On a test of visual resolution of a simulated structure with a linear density comparable to that of a labelled biological object (0.5 nm^−1^), the Voronoi visualization exhibits the best overall perception of the structure, discrimination between two closely located objects and complete suppression of noise (Figs 1A–E and S1). FRC curves calculated from Voronoi-density images lie at slightly higher frequencies than those of histogram- or Gaussian-mode images (Figs 1F and S1E–G). This resolution quantification indicates that the Voronoi-based visualization preserves the amount of useful information upon image rendering; this could be related with the fact that Voronoi-based reconstructions provide a better continuity of the signal (Fig. 1C), notably as compared to histogram-based reconstructions (Fig. 1A), which leads to a stronger correlation in FRC calculations.

Next, we extended the concept of Voronoi diagrams to perform cluster analysis, taking advantage of the intrinsic presence of Voronoi tessellations during the image reconstructions process (Fig. 2). A cluster is generally defined as a set of objects that are more similar to each other compared to objects in other sets. The properties of Voronoi cells (morphometric parameters such as shape, surface area, eccentricity etc.^19^,^23^) can be used as criteria for the association of several cells into clusters. We used intensity and spatial proximity parameters as determinants for clustering, allowing closely located fluorophore locations to be grouped according to the local fluorophore density. Thus, we define a cluster as a collection of neighboring Voronoi zones with areas smaller than a given threshold.

To test whether an experimental data set is significantly different from a spatially random distribution that has fluctuations of local density for a finite number of points, we performed Monte-Carlo simulations of randomly distributed points through the same experimental area using the same average density (Fig. 2; see methods). The probability distribution functions were calculated for experimental and for randomized data (including the mean value and associated 95% confidence interval; Fig. 2D). The comparison of distributions of Voronoi cell sizes of clustered and randomly distributed datasets reveals three characteristic regions (Fig. 2D): (i) small cell areas with an occurrence higher than in the random data set (cells describing clusters), (ii) intermediate regions with lower occurrence (containing Voronoi cell sizes similar to those of randomly distributed data), and (iii) regions of large Voronoi cells with higher occurrence corresponding to low-density event regions between clusters. The intermediate region (ii) can serve to define the boundaries of the clusters (i). The first intersection between the two density probability distribution functions (Fig. 2D) corresponds to the maximal cell size in the clusters, and this value can be used as a threshold value to automatically define regions of clustered polygons. Furthermore, calculation of the total area of the clustered polygons provides a direct measure of cluster occurrence which allows quantification of the fluorescence events within a given region. The inverse value of the obtained threshold can be used for binarization of the interpolated density map producing results similar to segmentation of Voronoi diagram (Fig. 2E,F). The difference is that clusters originated from the density map have smooth borders as the underlying Voronoi polygons are not displayed (Fig. 2F).

We applied the Voronoi tessellation strategy to visualize and quantify clustering observed in several important biological objects. A first example is the imaging of nuclear pore complexes (NPC; Fig. 3). Using Voronoi tessellation, individual NPCs (with a measured diameter of 114 ± 22 nm which corresponds to its known size^24^) can be distinguished even from sparse labeling (Fig. 3C,D), while this would not be obvious visually from a histogram-based representation (Fig. 3B). The quantification shows that an average density of ~5.4 NPC complexes per μm^2^ is found which is consistent with previous data^24^,^25^.

We next extended the Voronoi tessellation method to multi-color super-resolution data to address the possibility of co-localization analysis (Fig. 4). For segmented multi-color images the co-localization value *S/S*_*i*_ for a given species *i* is defined by the ratio of the superposed area *S* between two colors relative to the total area *S*_*i*_ of clusters of a given species; this value is further compared with the confidence range for randomly distributed clusters *S*_*rand*_*/S*_*i*_. Thus, an *S/S*_*i*_ value in the interval from 0 to the lower boundary of the confidence range means anti-localization or mutual exclusion, and a value in the interval from the upper boundary of the confidence range to 1 corresponds to co-localization. To demonstrate the applicability of our co-localization method to a biological object we used tubulin simultaneously labeled with two dyes (Alexa Fluor-555 and Alexa Fluor-647). The Voronoi tessellation diagram calculated for each color mode separately or merged shows clusters along the microtubule fibers (Fig. 4A). For the tubulin sample, we observed relatively high co-localization values (S/S_red_ = 0.416, S/S_green_ = 0.405) compared to a random distribution (S_rand_/S_red_ = 0.123 ± 0.021, S_rand_/S_green_ = 0.120 ± 0.020) which confirms the visual impression of overlapping fiber structures. This, together with the simulations of different co-localization scenarios (Fig. S2), shows the validity of the Voronoi tessellation approach for co-localization analysis, as an alternative to coordinate-based methods^6^,^7^.

To test the performance of Voronoi tessellation on dual-color labeling of different proteins we acquired super-resolution data on the cell nucleus (Fig. 4B) with RNA polymerase labeled with Alexa Fluor-488 and histone protein H2B labeled with Alexa Fluor-647. Interestingly, RNA polymerase is found more clustered in different regions of the cell nucleus (Fig. 4C), while histone H2B distribution (as compared to RNA polymerase) is more random within a given region (as illustrated by a shift of the density distribution compared to the random distribution function, Fig. 4E). The clustering analysis and quantification using the Voronoi cell areas show that histone H2B labeling exhibits very weak co-localization with RNA polymerase II (S/S_Pol_ = 0.090; S/S_H2B_ = 0.106), equivalent to uncorrelated simulated data (S_rand_/S_Pol_ = 0.096 ± 0.013; S_rand_/S_H2B_ = 0.113 ± 0.015). Figure 4C shows that the clusters of the two proteins do not overlap. This observation appears consistent with the idea that DNA transcription by RNA polymerase occurs at H2A/H2B-depleted regions of less tightly compact chromatin^26^,^27^,^28^, but this particular aspect would need to be analyzed in more detail in future studies, for example by H3 or H4 labeling. The Voronoi tessellation reveals an interesting non-random distribution of RNA polymerase molecules with a nearest neighbor distance between clusters (d = 76 ± 32 nm). This suggests that they form some sort of superstructures which could correspond to actively transcribing regions in the cell nucleus.

An important property for super-resolution microscopy of Voronoi diagram is its geometric stability with respect to small changes of its sites^29^, meaning that imprecise molecular localizations do not strongly influence results of segmentation. Indeed, we observed full retrieval of the number of clusters and their size in data with localization error σ_loc_ 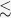 3 · *δ*^−1/2^ and visually recognizable clustering with σ_loc_ 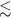 6 · *δ*^−1/2^ (estimated empirically) where σ_loc_ is the standard deviation of localization precision and *δ* is the density of clustered molecules (Fig. S3).

Multiple localizations of the same fluorescent molecules (through re-activation, blinking or multiple antibodies/fluorophores) may affect cluster analysis. By testing different simulated conditions we show that multiple localization (for example 2–6x more points distributed around a localization event) can improve the robustness of the segmentation under conditions of low background density (Fig. S4A) because more points are being used for defining clusters (*i.e.* the Voronoi cells become smaller and clusters show up better). In contrast, re-localizations of clusters with strong background lead to detection of spurious clusters in background regions (assuming multiple localization both for clustered points and for the background; Fig. S4B). Multiple localizations of randomly distributed points demonstrate false positive results in the Monte-Carlo simulations (Fig. S5), but if the number of re-localizations is known, it can be incorporated into the simulations leading to the correct results both in the evaluation of distributions for randomness and in segmentation (Fig. S5B,C).

Taken together, we present a robust method based on Voronoi tessellation for the convenient visualization and quantification of the localization and distribution of fluorescently labeled complexes, which allows segmentation, cluster analysis and estimation of the amount of co-localization. We show that image reconstruction using Voronoi diagrams preserves resolution equally well or better than Gaussian or histogram modes at the level of image rendering, as quantified by FRC. Voronoi tessellation allows performing a statistical analysis of the clusters, their occurrence and inter-cluster distance distribution, and works well including for the analysis of weak signals. Indeed, the general applicability of the method is illustrated here on a series of important biological objects including chromatin complexes, RNA polymerase, NPCs and microtubules. One of the advantages of the Voronoi tessellation method is that it does not require any *a priori* knowledge for the clustering (e.g. user-defined parameters such as the search radius for Ripley’s analysis, the number of clusters in the case of k-means clustering or search distance and number of points for DBSCAN; these methods do not assign an area of influence to data points such as Voronoi tessellation does which also provides boundaries between clusters). Because the clustering uses an internal reference generated from randomized data to automatically determine the threshold value for forming clusters between neighboring Voronoi zones, it is fully automated for a given region of interest. We have implemented the Voronoi tessellation method as a ClusterViSu standalone software which can also be interfaced as a plugin with an interactive open-source software for processing of super-resolution fluorescence microscopy data (SharpViSu; ref. 30; https://github.com/andronovl/SharpViSu) which includes other standard tools such as corrections for drift and chromatic aberration and resolution estimation by FRC. In the future, the approach could be extended to 3D super-resolution data because Voronoi diagrams are also defined for multi-dimensional cases.

To our knowledge, this is the first application of Voronoi tessellation for the clustering analysis of super-resolution microscopy data, together with a related method published by Levet *et al.*^17^ while the present manuscript was under review. To quantify differences between ClusterViSu and SR-Tesseler we run some simulations (see methods; Fig. S6). When compared with SR-Tesseler^17^, our method shows a more complete detection of the cluster numbers and retrieval of their size and homogenous shape (Fig. S6A–E), especially at conditions of weak density of clustered points or weak background (Fig. S6C–E). In ClusterViSu, the detection of clusters is insensitive to background densities, while SR-Tesseler shows some artefacts for weak densities (Fig. S6E). The segmentation threshold determined automatically by Monte-Carlo simulations as proposed in our work is more stable over a large range of background densities (Fig. S6F) as opposed to thresholds determined by the average localization density^17^. In ClusterViSu, the threshold diminishes slightly with increasing background which favorably reduces spurious detection of clusters (Fig. S6F), while the increasing threshold values can lead to under/overestimation of the cluster number at low/high densities in SR-Tesseler. The usage of complete spatially random distributions of the background in our study as opposed to uniform distributions^17^ provides more realistic simulations and explains the occurrence of spurious clusters that contain small numbers of events (in ClusterViSu these can be removed with a filter). ClusterViSu uses zero-rank density calculations, while SR-Tesseler uses first-rank as defined in ref. 17 which is more resistant to false detections at high cluster and high background densities (Fig. S6D) due to averaging of neighboring Voronoi polygons. ClusterViSu is also compatible with multiscale segmentation^17^ (further segmentation of detected clusters), which can be useful for sub-classification of cellular structures. Additional differences are that our implementation already allows double-labeling and quantitative co-localization analysis using Voronoi-tessellation (Fig. 4) which is an important feature for super-resolution studies. In addition, we quantify the resolution of image rendering using Voronoi diagrams and show that it is at least as good as that of Gaussian and histogram mode reconstructions. Furthermore, the four biological examples that we give in this work extend the field of applications of Voronoi-based tessellation, segmentation and quantification of data.

## Methods

### Super-resolution imaging

**Figure 1:**
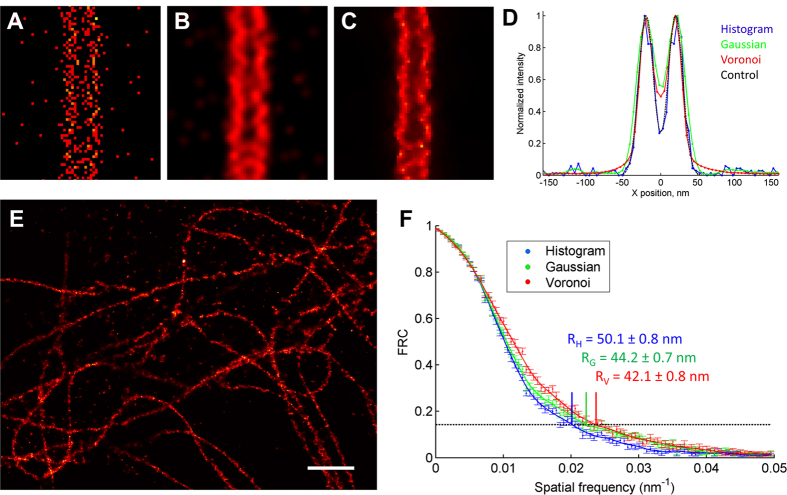
Comparison of different representation methods of localization events in super-resolution microscopy, showing that image representation by Voronoi diagrams fully preserves the resolution. (**A**–**C**) Simulated images of two lines separated by 40 nm and composed of localization events with a standard deviation of 10 nm in histogram (**A**), Gaussian (**B**) and Voronoi-based interpolated (**C**) local density representations. (**D**) Corresponding image projections generate back the underlying line structure – sum of two Gaussians (black dashed line); for line profiles see Fig. S1D. Each graph is normalized on its peak value; pixel size is 5 nm. (**E**) Voronoi density map of β-tubulin detected with Alexa-647 conjugated secondary antibodies. Pixel size is 10 nm, scale bar is 1 μm. (**F**) FRC curves calculated from a larger image containing (**E**) in histogram (blue), Gaussian (green) and Voronoi (red) representations with corresponding resolutions R_H_, R_G_ and R_V_ obtained by the 1/7th FRC criterion, showing that the best resolution is obtained by Voronoi representation. The dataset used for calculation of FRC contained 2.3 · 10^5^ localizations, the images in the three representations were reconstructed using a pixel size of 10 nm.

HeLa cells were plated in a 4-compartment glass-bottom petri dish (CELLView, Greiner Bio-One) and fixed with 4% formaldehyde for 20 min in phosphate-buffered saline solution (PBS). After permeabilization with 0.1% Triton in PBS (PBS/Tx) twice for 10 min, the primary antibody (anti-β-tubulin monoclonal (1Tub-2A2, in house IGBMC) was used as mouse ascites fluid diluted 500× in PBS/Tx; RNA Pol II monoclonal antibody (1PB-7C2, in house IGBMC), directed against the CTD of the largest subunit of RNA Pol II (RPB I) was used as a purified IgG at 5 μg/ml in PBS/Tx; histone H2B monoclonal antibody (LG11-2) was used as a 500× dilution of mouse ascites fluid in PBS/Tx) was incubated overnight at 4 °C. The sample was then washed with PBS/Tx three times over 2 hours, and the secondary antibody (goat anti-mouse Alexa Fluor-647 or Alexa Fluor-555 conjugated, Invitrogen) in dilution 4 μg/ml in PBS/Tx was incubated for 2 hours at room temperature. Subsequently, the cells were washed in PBS/Tx three times for 2 hours, then briefly three times in PBS. For the TPR sample, cells were cultured on coverslips and fixed in 4% paraformaldehyde for 10 min, permeabilized in 0.5% Triton for 10 min, blocked in 1% BSA for 30 min, and incubated with primary antibodies (TPR rabbit polyclonal antibody, Abcam, ab84516) for 1h and with secondary antibodies (goat anti-rabbit Alexa Fluor-647) for 45 min^14^. The double-labelled β-tubulin sample was mounted in an imaging buffer^31^ that contained 20% of Vectashield (Vector Laboratories), 70% of 2,2′-thiodiethanol (also known as thiodiglycol or TDE) and 10% of PBS 10× (the measured refractive index of this mounting medium is 1.491). The H2B/RNA Pol II, the TPR and the β-tubulin sample used for Fig. 1 were mounted in a PBS buffer with addition of 10 mM of cysteamine (also known as β-mercaptoethylamine or MEA) and 25 mM of HEPES (pH 7.5).

The super-resolution experiments were performed on a Leica SR GSD system built on a base of DMI6000 B inverted wide-field microscope. We used the HCX PL APO 100×/1.47 Oil CORR TIRF PIFOC objective with a 1.6× magnification lens that provides an equivalent pixel size of 100 nm on the Andor iXon3 DU-897U-CS0-#BV EMCCD camera with a field of view of 18 × 18 μm in super-resolution mode. Continuous wave fiber lasers (MPBC Inc., 488 nm 300 mW, 532 nm 1000 mW, 642 nm 500 mW) and a diode laser (405 nm 30 mW) were utilized for excitation. The microscope is also equipped with a suppressed motion (SuMo) sample stage, which reduces drift but does not eliminate it (typical values 20–50 nm over 10 min). The residual drift was corrected by data processing (see below).

The samples were first illuminated with the 100% power of the appropriate laser to quickly send the fluorophores into the dark state. The acquisition started after beginning of observation of single-fluorophore events (“blinking”) that corresponded to the drop of the correlation value of consecutive frames to approximately 0.2 in the corresponding wizard in the LAS AF software. The time of exposition of a frame was 6.34 ms (H2B and RNA Pol II data) or 50 ms (β-tubulin and TPR data); the electron multiplying gain of the camera was 300 for H2B, RNA Pol II, TPR, 150 for β-tubulin-Alexa 647 and 63 for β-tubulin-Alexa 555; the laser power during the acquisition was 30% (H2B), 50% (TPR) or 100% (RNA Pol II and β-tubulin). After a few minutes, as the number of blinking evens dropped, the sample started to be illuminated additionally by a 405 nm laser with gradual increase of its intensity in order to keep a nearly constant rate of single-molecular returns into the ground state. The acquisition was stopped after almost complete bleaching of the fluorophore.

### Data processing

The localization and fitting of single-molecule events were performed in real time during acquisitions in Leica LAS AF 3.2.0.9652 software with the “center of mass” fitting method. To reduce the number of localizations of the same fluorophore and improve localization precision the data were processed by averaging the coordinates of consecutive events within a radius of 50 nm around each localization. Subsequently, the data were processed in SharpViSu (ref. 30, in press; https://github.com/andronovl/SharpViSu). The drift was detected and corrected using cross-correlation-based approach. Briefly, the dataset was divided on several consecutive subsets, from each of them a histogram image with pixelation of 20–40 nm was build, the shift between these images was detected with subpixel precision and interpolated linearly throughout intermediate frames. The shift was then subtracted from every frame. The procedure was repeated iteratively 4 times assuring absence of detectable residual drift. For correction for the chromatic aberrations, the aberrations were detected beforehand with Tetraspeck multi-color beads, interpolated through the field of view with 2-order polynomial functions and subtracted from the molecular coordinates obtained from either 488 nm or 532 nm imaging channels. Regions of interest (ROI) for Voronoi analysis were selected manually allowing faster computations and more homogeneous distributions compared to the entire field of view.

**Figure 2:**
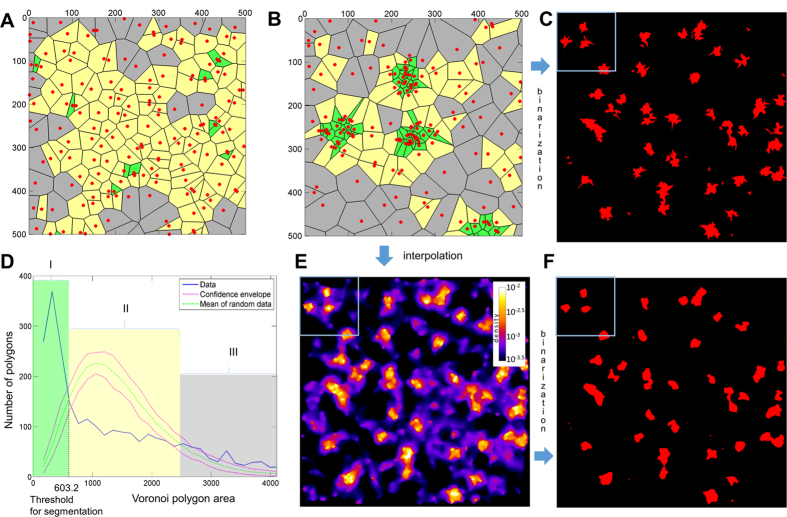
Principle of Voronoi-based image segmentation which allows visualization and quantification of clusters. (**A**) A region of simulated pointillist data with random distribution. (**B**) Simulated clustered distribution of the same number of points as in the random dataset. (**C**) Clustering obtained after direct segmentation of the Voronoi diagram allowing visualization of the clusters. (**D**) Distribution of Voronoi polygon areas of the clustered dataset (blue) and mean Voronoi polygon distribution of a random dataset (green) with confidence envelope (red) obtained from Monte-Carlo simulations which allows defining a threshold value for automated segmentation. The three characteristic regions: small clustered polygons (I, green); intermediate polygons corresponding to the random distribution (II, yellow); huge polygons corresponding to background in the clustered distribution (III, gray). (**E**) Interpolation of the local densities to pixel grid produces local density map. (**F**) Clusters, obtained by thresholding of the density map. The simulated dataset contained 48 clusters with a radius of 50 nm consisting of ~27 events each. After segmentation, the Voronoi tessellation allows quantification of the clusters (35 were detected, excluding small clusters that contained only one localization event, with 29 ± 16 events each and with equivalent radius of 49 ± 11 nm). The blue frames in (**C**,**E**,**F**) correspond to the region shown in panel (**B**).

The following analysis was performed in Matlab using customized code that is included as modules in the ClusterViSu software. Voronoi diagrams (vertices of polygons and connectivity order) were retrieved with either ‘voronoi’ or ‘voronoin’ functions. Areas of the cells were determined from the vertices with the function ‘polyarea’, the local density in each data point was defined as the inverse value of the area of the corresponding Voronoi polygon. To have a smooth appearance that can be used for visualization (Fig. 1C–E) or segmentation (Fig. 2E,F) the values of the local density were interpolated to a regular grid (pixels) using the ‘griddata’ function and the ‘natural’ interpolation method^32^. The spacing of the grid corresponds to the desired pixel size (in range of 2 to 20 nm in our case, it is indicated in the figure legends; at least 3–4 times smaller than the expected resolution to satisfy the Nyquist theorem).

For the comparison of visualization techniques, we used a Gaussian distribution of points arranged as two parallel lines, with a distance of 40 nm between the centers of the distributions and the standard deviation of each distribution σ = 10 nm in the X direction (Fig. 1A–D). The linear density of localization in each line was 0.5 nm^−1^. To this dataset we added localizations with random (x, y) coordinates, with overall density of 400 μm^−2^, comparable to background density at experimental data. The photon counts for all events were distributed with Gaussian distribution around the mean value of 1000 photons with standard deviation of 300. This is similar to a procedure described by Baddeley *et al.*^4^ with following modifications: linear densities were chosen to be similar to those of typical experimental data, noise was added, and single line profiles (Fig. S1D) were used along with projections (Fig. 1D; even though projections tend to not reflect discontinuities in the data, i.e. they generate back the underlying line structure). The Gaussian mode image was built representing each event as a Gaussian filter kernel with σ = A/(N_ph_)^1/2^, where A = 240 nm (experimentally determined value for our system), and N_ph_ is the number of photons. The FRC curves were calculated in 90 concentric rings (resulting in 90 frequency values in the FRC graphs) using the corresponding type of image representation for half-datasets^3^. For statistics, the FRC curves and the resolution values were calculated 50 times for each localization table, using different random separations of the dataset on two parts. Standard deviations of the obtained values are shown in error bar for each frequency point. The FRC curves for histogram, Gaussian and Voronoi representations were also calculated by the FSC program (Image Science Software GmbH)^33^ confirming the results obtained with our method.

The simulated cluster data on Fig. 2 were generated as randomly distributed points in circles with a radius of 50 nm. The density of points in the clusters was 3 · 10^−3^. The positions of clusters and of low-density (4 · 10^−4^) background points were distributed randomly in the field of view (FOV). The distributions of the sizes of the Voronoi cells were built as histograms of the areas of the cells, for a range from 0 to 4 times the median value of the area, using 2N^1/3^ bins, where N is the number of the polygons, excluding infinity-sized polygons at the boundaries of the ROI. For Monte-Carlo simulations, random coordinates were generated throughout the ROI using the ‘rand’ function. To obtain the confidence envelope, the distributions were generated for 50 different random sets of points, the boundaries of the envelope were determined as <n> ± 1.96 σ for each bin of the histogram, where <n> is the average number of cells within the range of the bin and σ is the standard deviation of n, calculated from the 50 random datasets. The abscissas of the first and the second intersections between the curves of the experimental and the mean value of the randomized distributions were determined from the two points around the intersections in the linear approximation. The Voronoi cells smaller than the area corresponding to the first intersection were kept, and all touching cells (those that have at least one common vertex) were combined together yielding vertices of clusters. The clusters were rasterized with the ‘poly2mask’ function using a pixel size of 1 nm. For the nuclear pore data a threshold was used to set the minimum number of events to remove small, spurious clusters. In our current implementation calculations for medium-sized datasets, e.g. Monte-Carlo simulations of 5⋅10^4^ localizations, take around a minute; calculations may be parallelized in future software versions.

The simulated data on Figs S3–S6 were generated as 10 × 10 clusters with radius of 50 nm through a FOV of 4 × 4 μm. The positions of the centers of the clusters were distributed regularly in the FOV. The points inside the clusters and in background were placed randomly with the indicated average density. For Fig. S3 the positions of the points were picked from the normal distribution using the mean defined at the previous step and the standard deviation of σ_loc_. For Figs S4 and S5, n (number of re-localizations) points were picked for every original point using the normal distribution with the standard deviation σ_loc_ = 10 nm and the mean value at the position of the original point. For Monte-Carlo simulations on Fig. S5 the randomized datasets were formed using N/n_sim_ seeds at spatially random positions and distributing n_sim_ points by Gaussian distribution with σ_loc_ = 10 nm around the seeds; N is the number of points in the original dataset, n_sim_ is the number of re-localizations used in the Monte-Carlo simulations.

For segmented multi-color images, the co-localization value for a given species is defined by the ratio of the superposed area S between two colors relative to the total area of clusters of a given species. To compare the obtained values with overlapping area S_rand_ of randomly distributed clusters we shuffled the experimentallydetermined clusters into randomized positions through the same field of view, independently for each color. Firstly, the list of cluster polygons was permutated randomly using Matlab’s ‘randperm’ function. Then, we determined the centroids of the clusters by formulas: 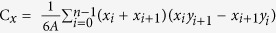, 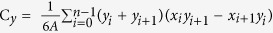; where C_x_, C_y_ are the (x, y) coordinates of the centroid of a given polygon, x_i_, y_i_ are the coordinates of the vertices, n is the number of the vertices, and the vertex i = 0 equals to the vertex i = n. A is the signed area of the polygon: 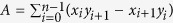. After subtraction of the centroid coordinates from coordinates of the vertices of the polygons, the new coordinates were determined as a random number situated in the FOV of the initial size. To avoid overlapping of newly placed clusters, we iteratively checked for each cluster being placed at a random spot if it was not overlapping with the previously placed clusters of the same color, in which case another random coordinate was chosen. The shuffling procedure was repeated 50 times with different random positions, and the 95% confidence range for the co-localization in the random case was obtained as mean ±1.96 σ of the corresponding ratios of the surfaces.

Pair distribution functions were calculated using Matlab’s ‘pdist2’. For the nearest neighbor distance, the smallest pairwise distance value was chosen for each data point. The equivalent radius of a cluster was calculated as the radius of a circle with the same surface area as the cluster. Quantified properties of clusters (number of events, equivalent radius, nearest distance between neighboring clusters) are represented as mean ± standard deviation of the corresponding values.

## Additional Information

**How to cite this article**: Andronov, L. *et al.* ClusterViSu, a method for clustering of protein complexes by Voronoi tessellation in super-resolution microscopy. *Sci. Rep.* **6**, 24084; doi: 10.1038/srep24084 (2016).

## Supplementary Material

Supplementary Information

## Figures and Tables

**Figure 3:**
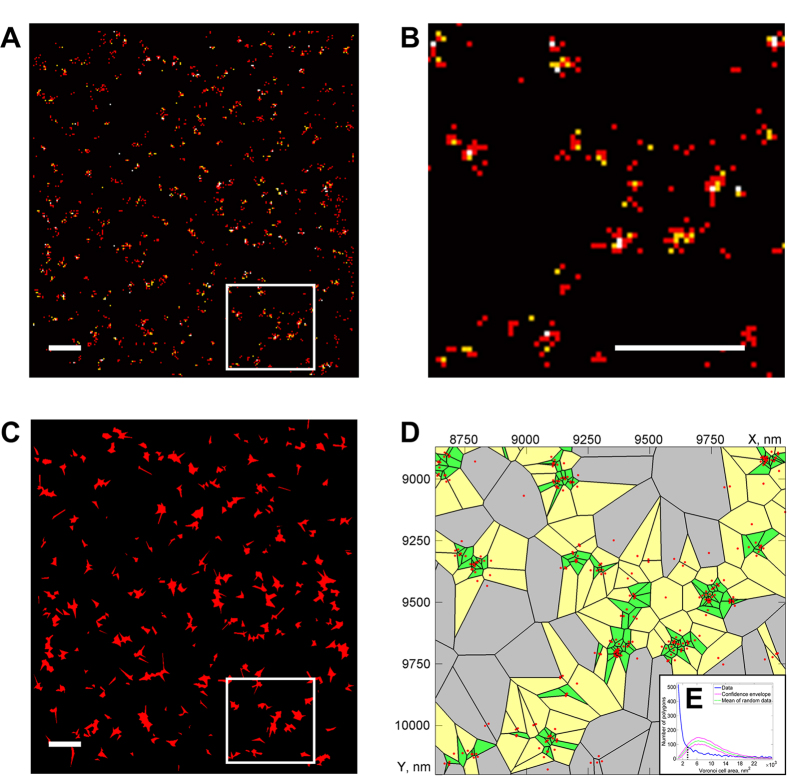
Segmentation and cluster analysis of nucleoporin protein TPR. (**A**) Histogram-based image reconstruction of TPR distribution at the nuclear envelope with a magnified region (white box) in (**B**). (**C**) Segmented Voronoi diagram calculated from the image in (**A**) with magnified region of the diagram shown in (**D**) which allows visualization and quantification of the TPR clusters present in the magnified region of the original image (panel **B**) in which quantification was not straightforward. (**E**) Voronoi polygon distribution of TPR (blue) demonstrating clustering as compared to random distributions (green and red). The clusters contain 9.7 ± 7.3 events with an equivalent radius of 57 ± 11 nm. Small clusters containing 2 or less events were excluded for this quantification. Scale bars correspond to 500 nm (**A**–**C**), pixel size is 20 nm (**A**,**B**).

**Figure 4:**
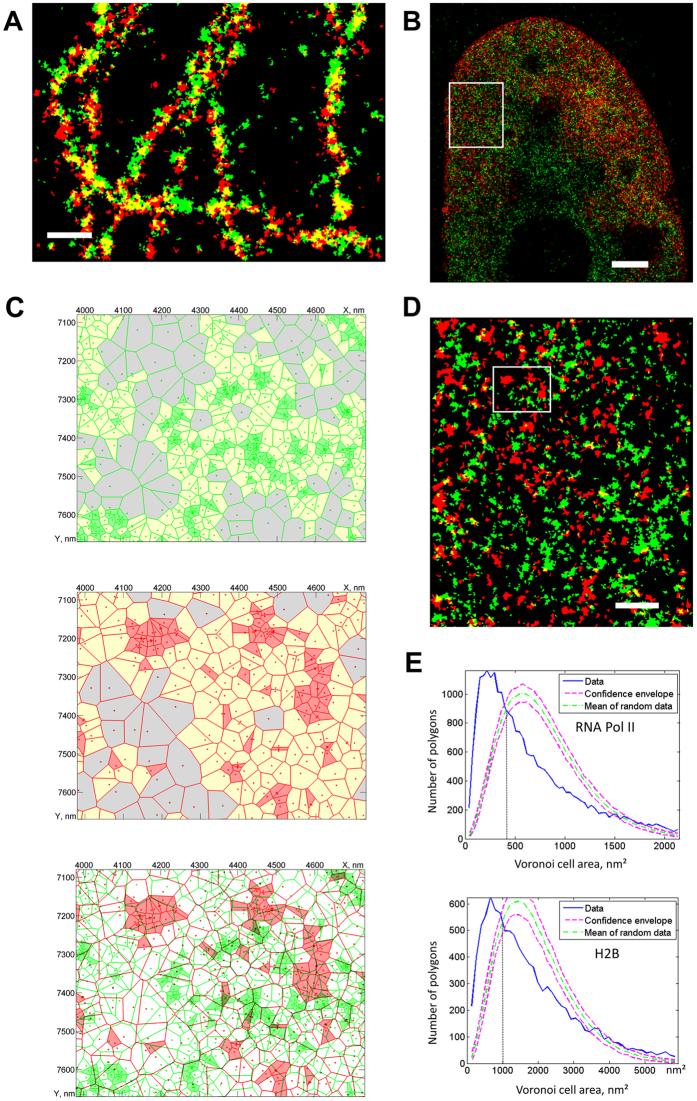
Co-localization analysis using Voronoi tessellations. (**A**) Segmented Voronoi diagram of β-tubulin, labelled with two different fluorophores (Alexa Fluor-555, green, and Alexa Fluor-647, red) demonstrating strong co-localization (S/S_red_ = 0.416, S/S_green_ = 0.405) compared to randomly distributed clusters (S_rand_/S_red_ = 0.123 ± 0.021, S_rand_/S_green_ = 0.120 ± 0.020). (**B**) A cell nucleus with labelled RNA Pol II (green) and histone H2B (red), represented as Voronoi local density map. (**C**) Example of Voronoi diagrams for RNA Pol II, H2B and their overlay, revealing no co-localization. (**D**) Segmented Voronoi diagram, used for determination of cluster properties (average cluster size: RNA Pol II 1818 nm^2^, H2B 3058 nm^2^) and for calculation of co-localization (S/S_Pol_ = 0.090, S/S_H2B_ = 0.106; random: S_rand_/S_Pol_ = 0.096 ± 0.013, S_rand_/S_H2B_ = 0.113 ± 0.015) revealing no correlation between the two distributions of clusters. (**E**) Voronoi polygon distributions of the clustered dataset (blue) and mean density distribution of the random datasets (green) with confidence envelopes (red) obtained from Monte-Carlo simulations for the RNA Pol II and H2B data. Scale bars 300 nm (**A**), 2 μm (**B**), 500 nm (**D**); panel (**D**) corresponds to the frame in panel (**B**) and (**C**) to that in panel (**D**).
