# Supplementary material for: ClusterViSu, a method for clustering of protein complexes by Voronoi tessellation in super-resolution microscopy
Source: Sci Rep. 2016 Apr 12;6:24084. doi: 10.1038/srep24084 (PMC4828638; doi:10.1038/srep24084)
Supplement: Supplementary Information [file srep24084-s1.pdf]

ClusterViSu, a method for clustering of protein complexes by Voronoi tessellation in super-resolution microscopy

Leonid Andronov[1,2,3,4], Igor Orlov[1,2,3,4], Yves Lutz[1,2,3,4], Jean-Luc Vonesch[1,2,3,4], Bruno P. Klaholz[1,2,3,4,*]

[1]Centre for Integrative Biology (CBI), Department of Integrated Structural Biology, IGBMC (Institute of Genetics and of Molecular and Cellular Biology), 1 rue Laurent Fries, Illkirch, France; [2]Centre National de la Recherche Scientifique (CNRS) UMR 7104, Illkirch, France; [3]Institut National de la Santé et de la Recherche Médicale (INSERM) U964, Illkirch, France; and [4]Université de Strasbourg, Strasbourg, France.

*To whom correspondence should be addressed. klaholz@igbmc.fr

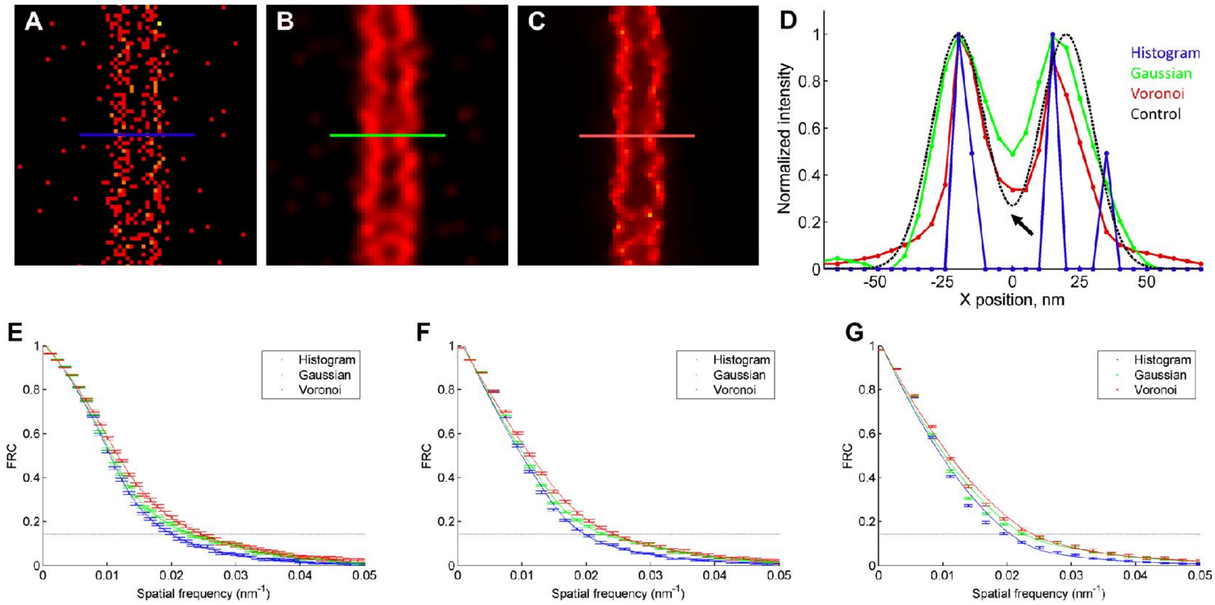

Figure S1 Evaluation of the quality of Voronoi image reconstruction

(**A-C**) Simulated images of two lines separated by 40 nm and composed of localization events with a standard deviation of 10 nm in histogram (**A**), Gaussian (**B**) and Voronoi-based interpolated (**C**) local density representations (same as **Figs. 1A-C**, but with line indicating the line profile). (**D**) One-pixel width profiles along the colored lines of images **A-C**. (**E-G**) FRC curves calculated from an image region in **Fig. 1E** in histogram (blue), Gaussian (green) and Voronoi local density (red) representations; pixel size of the image: 5 nm (**E**), 3 nm (**F**), 2nm (**G**). The graphs show only the frequency range from 0 to 0.05 nm^{-1} for consistency with **Fig. 1F**.

Suppl. Fig. S1

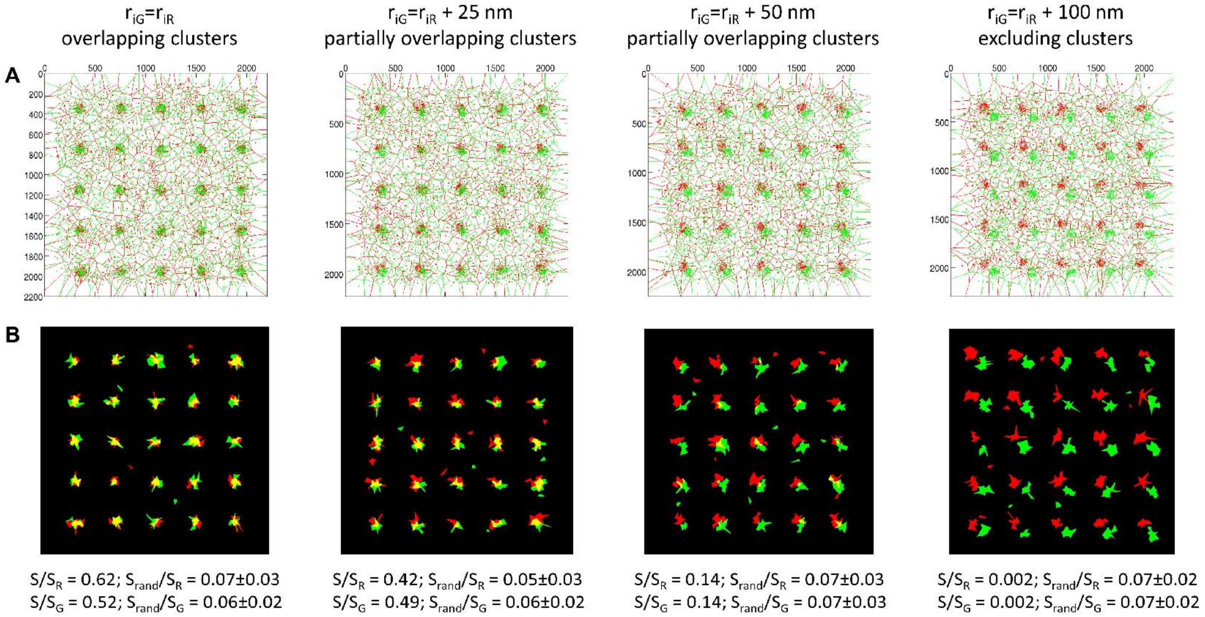

Figure S2 Simulations of different co-localization scenarios

(**A**) Voronoi diagrams of simulated double-color clusters. (**B**) Results of segmentation of the double-color clusters in ClusterViSu with the overlay area shown in yellow and the calculated co-localization values shown below. r_{iG} and r_{iR} are the Cartesian coordinates of the centers of the green and the red clusters, respectively. The simulated radius of clusters is 50 nm; density of events in clusters = $2.5 \cdot 10^{-3}$ nm^{-2}, in background = $1.25 \cdot 10^{-4}$ nm^{-2}. Localizations in the clusters and in the background are distributed randomly and independently for each color.

Suppl. Fig. S2

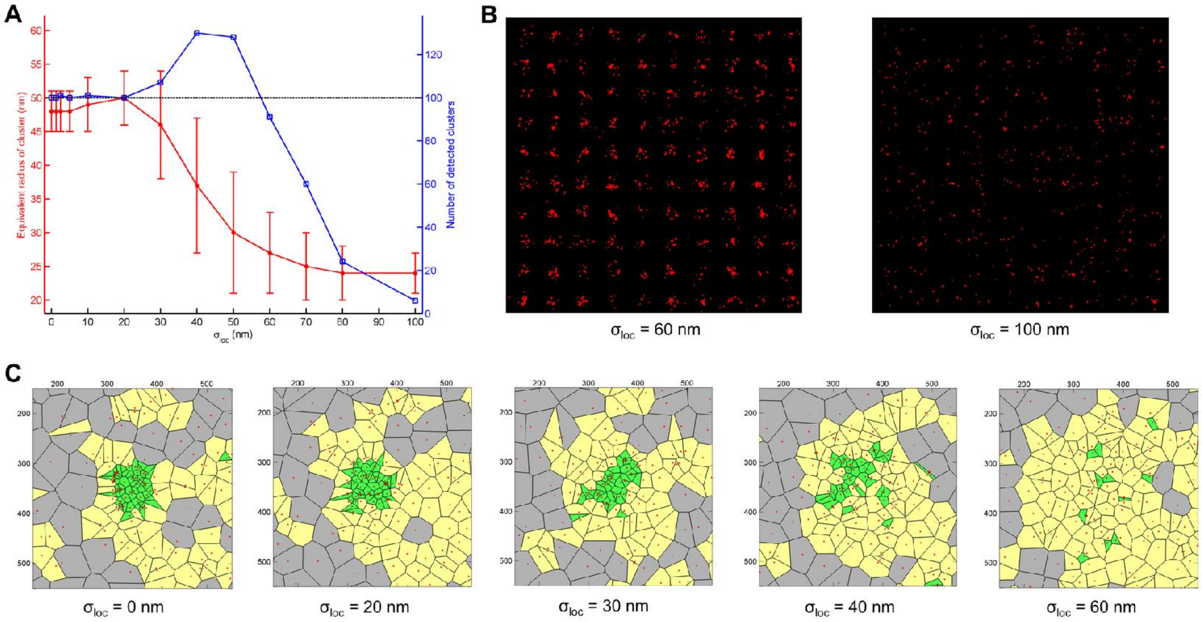

Figure S3 Effect of the localization error on Voronoi clustering

(**A**) Results of segmentation of a simulated cluster data with different amounts of localization error. σ_{loc} is the standard deviation of the normal distribution used for simulating the coordinates of the points. Clusters containing 5 or less molecules were removed. (**B**) Complete segmented FOVs of the simulated data with high localization errors σ_{loc} demonstrating remaining clusters at σ_{loc} = 60 nm and σ_{loc} = 100 nm. (**C**) The Voronoi diagrams of the upper-left corner of the simulated data with different localization errors. The density of clustered localizations is 0.01 nm^{-2}, the density of background is 0.002 nm^{-2}.

Suppl. Fig. S3

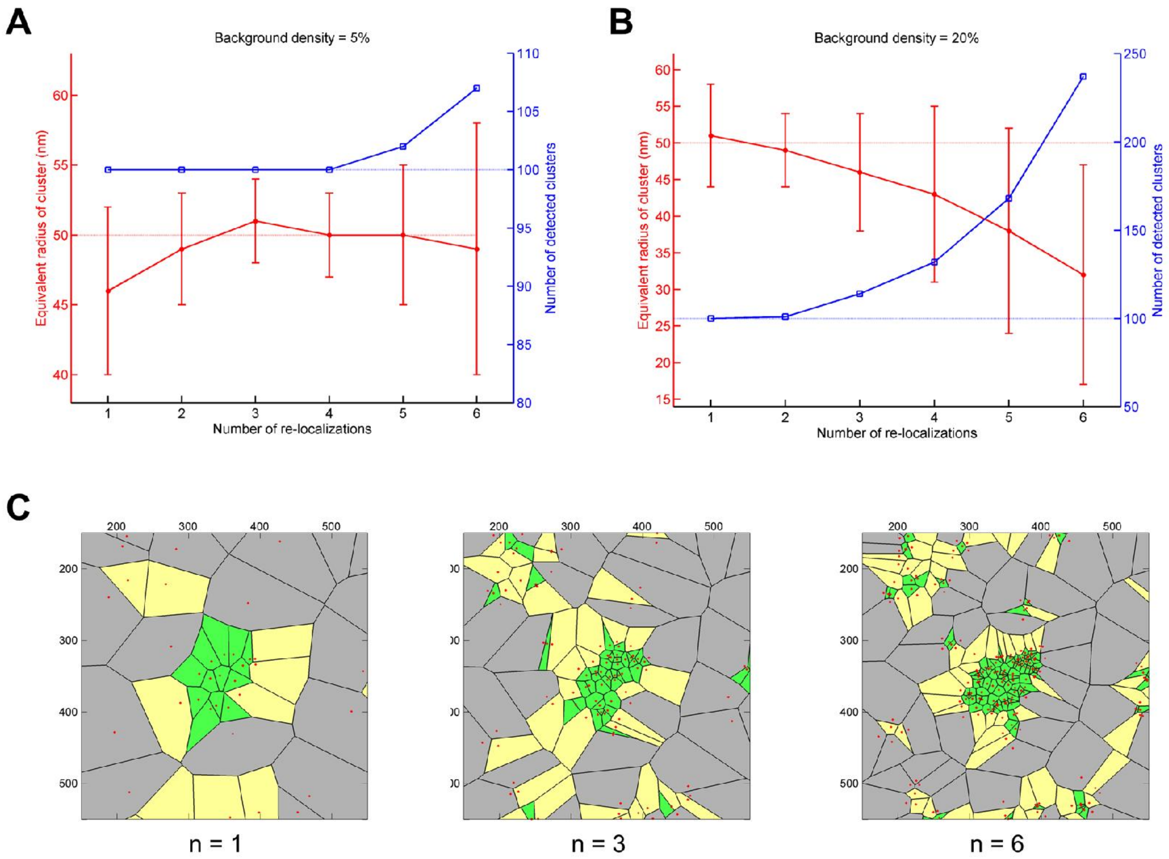

Figure S4 Effect of multiple localizations on Voronoi clustering

(**A&B**) Results of segmentation of simulated cluster data with different numbers of re-localizations. Clusters containing 5 or less molecules were removed. (**C**) Comparison of Voronoi diagrams with different numbers of re-localizations (n). The density of clustered localizations (at n = 1) is 0.0025 nm^{-2}), the density of background is 20% of the cluster density; the localization precision σ_{loc} = 10 nm.

Suppl. Fig. S4

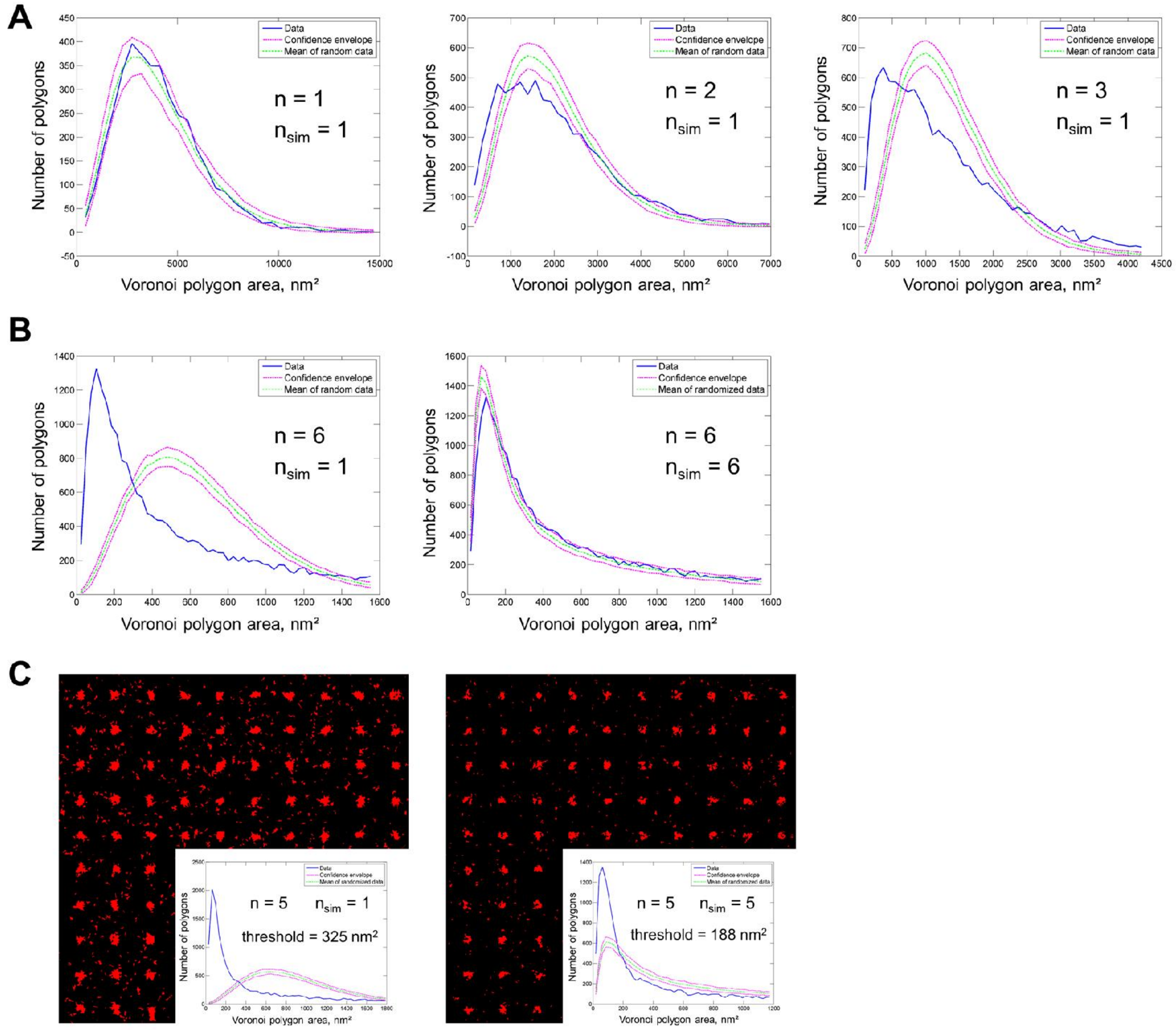

Figure S5 Effect of multiple localizations on Monte-Carlo simulations

(**A&B**) Voronoi polygon distributions of spatially random datasets with different numbers of re-localizations (n) and results of Monte-Carlo simulations taking into account multiple localizations (n_{sim}). (**C**) Simulated clustered datasets with re-localizations (n = 5) segmented by thresholds determined by simulations without (n_{sim} = 1) and with (n_{sim} = 5) multiple localizations. The density of localizations (at n = 1) is 0.001 nm^{-2} (**A&B**); the density of clustered localizations (at n = 1) is 0.0025 nm^{-2}, the density of background is 20% of the cluster density (**C**); the localization precision σ_{loc} = 10 nm (**A-C**).

Suppl. Fig. S5

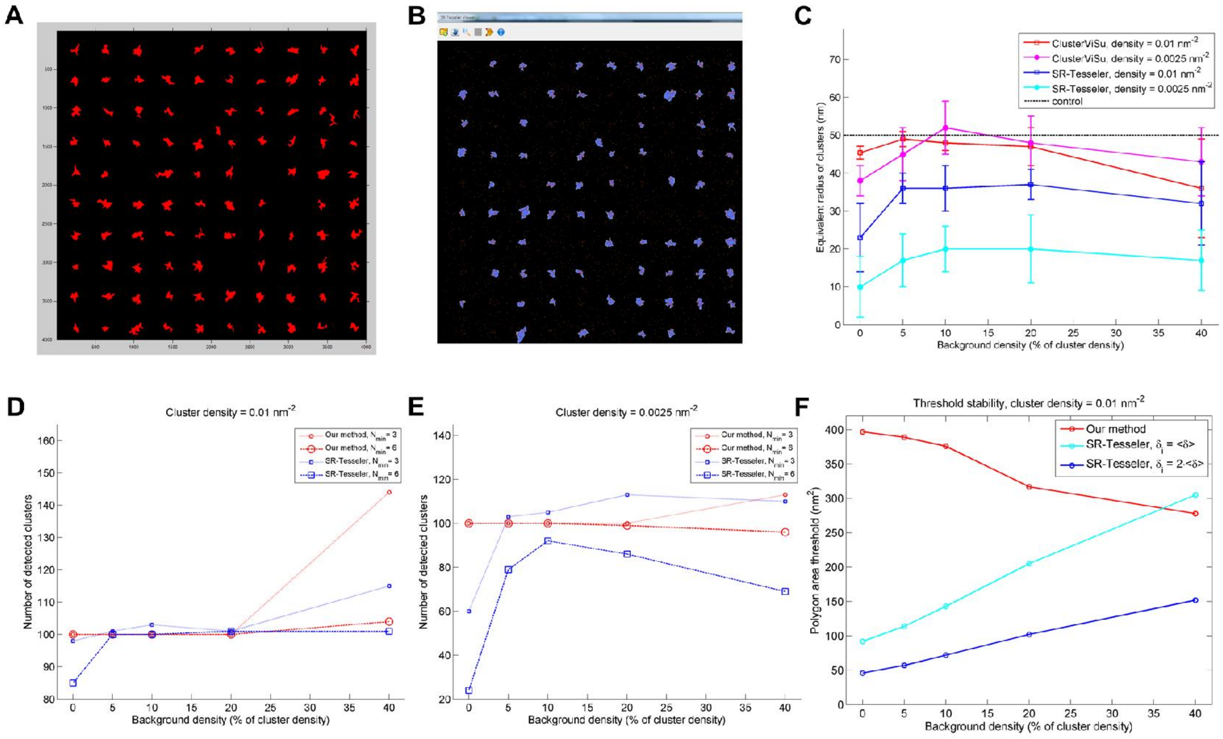

Figure S6 Comparison of ClusterViSu with SR-Tesseler.

(**A&B**) Clustered dataset segmented in ClusterViSu with threshold determined by Monte-Carlo simulations (**A**) and in SR-Tesseler with threshold determined by average localization density[17] $\delta^1_i = 2\cdot\langle\delta\rangle$ (**B**). Density of clustered localization is 0.0025 nm^{-2}, background density is 0.01 nm^{-2}. Clusters containing five or less molecules were removed. (**C**) Retrieval of the equivalent radius of clusters in ClusterViSu and SR-Tesseler. Clusters containing two or less molecules were removed. (**D&E**) Number of clusters detected by ClusterViSu (red curves) and SR-Tesseler (blue curves) as function of background density. N_{min}, minimal number of events in clusters kept for quantification. (**F**) Variation of segmentation threshold determined by Monte-Carlo simulations in ClusterViSu (red curve) or by average localization density in SR-Tesseler[17] (cyan and blue curves), depending on background density.

Suppl. Fig. S6
